# *Notes from the Field:* Case of Legionnaires Disease Associated with a Home Device Used to Mix Powdered Infant Formula — United States, 2025

**DOI:** 10.15585/mmwr.mm7523a1

**Published:** 2026-06-18

**Authors:** Eric J. Stern, Judie Hyun, Elizabeth J. Hannapel, Melisa Willby, Chris Edens

**Affiliations:** ^1^Division of Pediatric Infectious Diseases, Georgetown University Hospital, Washington, DC; ^2^Maryland Department of Health, Baltimore, Maryland; ^3^Division of Bacterial Diseases, National Center for Immunization and Respiratory Diseases, CDC.

**Summary**

**What is already known about this topic?**

Legionnaires disease is a serious pneumonia caused by inhalation or aspiration of *Legionella* bacteria. Stagnant, warm water (77°F–113°F [25°C–45°C]) increases the risk for *Legionella* growth.

**What is added by this report?**

Legionnaires disease was identified in an infant with an immunocompromising condition who had recently consumed formula prepared using filtered tap water and mixed by a home powdered formula preparation device. *Legionella pneumophila* serogroup 1 was detected in the household water and in higher concentrations in the formula preparation device.

**What are the implications for public health practice?**

Ready-to-feed formula may be used for formula-fed infants who have immunocompromising conditions; if powdered formula is used, it should be prepared with water heated to ≥158°F (≥70°C) and cooled before feeding. Infant formula mixing device manufacturers might consider revising their device instructions and designs to minimize the risk for *Legionella* bacteria growth.

On November 17, 2025, an infant girl aged 10 months was admitted to Georgetown University Hospital with fever, tachypnea, and chest retractions (Figure). She had previously been admitted 32 days earlier (October 16) for treatment of systemic-onset juvenile idiopathic arthritis macrophage activation syndrome, a life-threatening condition caused by uncontrolled activation of macrophages and T-cells. She had been discharged with immunosuppressant medications on November 5 and had remained stable at home. At routine follow-up outpatient visits on November 10 and 13, her condition was unchanged. While at home, she was fed formula prepared using a powdered infant formula mixing device. This type of device holds water and powdered formula and is designed to quickly dispense ready-to-drink, clump-free, warm formula. At the time of her November 17 hospital admission, although results from clinical testing for multiple pathogens* were negative, a chest radiograph showed left upper lobe consolidation. On November 21, a microbial cell–free DNA blood test (mcfDNA) (Karius Spectrum) was positive for *Legionella pneumophila*. On November 28, results from an *L. pneumophila* serogroup 1 urinary antigen test (UAT) (BinaxNOW *Legionella* urinary antigen card) were positive. No respiratory specimens were available for culture. Legionnaires disease primarily affects adults aged >50 years; cases in infants and children are rare (*1*). Infection is typically acquired through inhalation of aerosolized water, although infection from aspiration also occurs (*2*).

**FIGURE:**
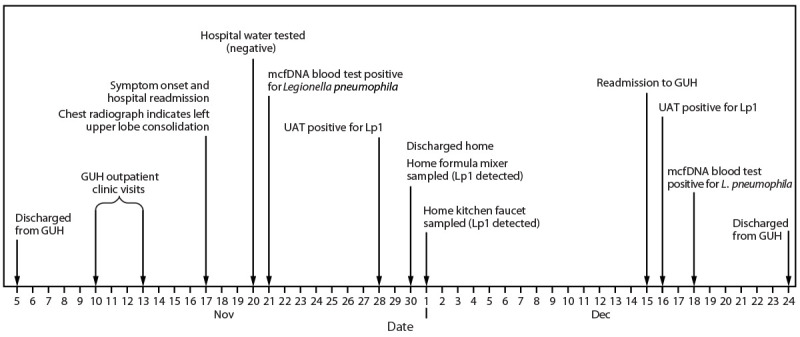
Timeline of Legionnaires disease diagnosis associated with a home device used to mix powdered infant formula — United States, November–December 2025 **Abbreviations:** GUH = Georgetown University Hospital; Lp1= *Legionella pneumophila* serogroup 1; mcfDNA = microbial cell–free DNA; UAT = urinary antigen test.

## Investigation and Outcomes

### Assessment of Potential Sources

Because the infant had health care exposures (recent hospitalization and outpatient visits) during the 14-day Legionnaires disease incubation period, potential health care and household sources were evaluated. This activity was reviewed by CDC, deemed not research, and conducted consistent with applicable federal law and CDC policy.^†^

Although routine hospital water testing on November 20 did not detect *Legionella* bacteria or abnormal chlorine values, the facility cannot be excluded as a source. No additional aerosol-generating exposures (e.g., humidifiers or showers) were identified at the home or health care facility. The infant’s primary potential exposure was formula prepared using a powdered infant formula mixing device (Baby Brezza Formula Pro Advanced Baby Formula Dispenser). This device stored water and powered infant formula in separate reservoirs. When activated, the device heated water to a user-specified temperature (using fixed presets), mixed in the powder, and dispensed ready-to-drink formula. 

### Assessment of Powdered Formula Mixing Device

Water from the powdered formula mixing device’s internal reservoir collected on November 30 tested positive for *L. pneumophila* serogroup 1 (72.5 CFU/mL) using traditional spread-plate culture. Water collected the next day from the kitchen faucet used to fill the device was also positive for *L. pneumophila* serogroup 1 (0.7–3.0 CFU/mL). This faucet had an under-sink filtration system that removed chlorine. The internal device reservoir contained water at 106°F (41°C); the parents reported they had not fully emptied and drained the device in >30 days, conditions conducive to *Legionella* species amplification (*1*).

### Hospitalization

On admission, the infant required oxygen via nasal canula and received cefepime, a broad-spectrum fourth-generation cephalosporin and vancomycin. After receipt of the mcfDNA *Legionella* test results on November 21, treatment was changed to azithromycin and meropenem. Her condition improved after a 5-day course, and she was discharged on November 30 on a 21-day course of oral levofloxacin. However, she was readmitted on December 15 with poor oral intake and chest radiograph findings showing new left lung cavitations. The family reported that they had discontinued use of the formula mixing device and had only used boiled tap water for formula preparation after November 30. Repeat UAT (December 16) and blood mcfDNA (December 18) tests were again positive for *L. pneumophila* serogroup 1 and *L. pneumophila*, respectively. Given the absence of new exposures and consistent detection of the same organism, these findings were interpreted as persistence of the initial infection. The infant recovered after a 6-day course of azithromycin and placement of a nasogastric tube and was discharged on December 24 to complete additional intravenous antibiotics at home (ceftriaxone for 20 days and clindamycin for 7 days). The health care provider submitted a Consumer Product Safety Commission report regarding this device on February 15, 2026.

## Preliminary Conclusions and Actions

A confirmed case of Legionnaires disease was diagnosed in an infant with an immunocompromising condition based on positive UAT results. Investigation identified *Legionella* bacteria in an infant formula mixing device and in the filtered household water used to fill the device as plausible contributors to infection via aspiration. The household water filter was not tested, although these devices might contribute to the growth of organisms if not properly maintained (*3*); removal of chlorine by the filter could have contributed to the growth of *Legionella *organisms. Limitations included lack of clinical and environmental isolates for comparison, the occurrence of possible health care exposures during the incubation period, and environmental sampling occurring approximately 2 weeks after illness onset.

The device manufacturer recommends using distilled or boiled water but does not specifically recommend routine internal reservoir draining. Storing water at 77°F–113°F (25°C–45°C) can promote *Legionella* bacteria growth (*2*). The device’s internal reservoir temperature (106°F [41°C]) and prolonged water storage likely facilitated bacterial amplification.

Household devices that retain warm water are possible *Legionella* bacteria sources and might pose a health risk, especially for persons with immunocompromising conditions. For infants with immunocompromising conditions who are fed reconstituted powdered formula, water should be heated to ≥158°F (≥70°C) before mixing to reduce risk for exposure to or infection with other bacteria in the formula (e.g., *Cronoboacter*). Prepared formula should be cooled before feeding to prevent scalding (*4*). Boiling water will kill *Legionella *organisms if they are present. Manufacturers might consider revising their device design and instructions to reduce the risk for bacteria growth by recommending additional routine reservoir maintenance and emphasizing risks associated with use of unboiled tap water to prepare infant formula, especially for infants with immunocompromising conditions.

## Footnotes

* Respiratory multipathogen polymerase chain reaction (PCR) panel (adenovirus, *Bordetella pertussis*, *Chlamydia pneumoniae*, Coronavirus_229E, Coronavirus_HKU1, Coronavirus_NL63, Coronavirus_OC43, SARS-CoV-2, human metapneumovirus, human rhinovirus/enterovirus, influenza A, influenza A H1, influenza A H3, influenza A H1N1/pdm09, influenza B, *Mycoplasma pneumoniae*, parainfluenza 1–4, and respiratory syncytial virus), blood culture, fungal blood culture, methicillin-resistant *Staphylococcus aureus* nares swab, *Aspergillus galactomannan* antigen, Fungitell (1,3)-beta-D-glucan assay, *Streptococcus pneumoniae* urinary antigen test, and cytomegalovirus and adenovirus plasma PCRs.

† 45 C.F.R. part 46.102(l)(2), 21 C.F.R. part 56; 42 U.S.C. Sect. 241(d); 5 U.S.C. Sect. 552a; 44 U.S.C. Sect. 3501 et seq.

## References

[1] CDC. *Legionella* (Legionnaires’ disease and Pontiac fever): how *Legionella* spreads. Atlanta, GA: US Department of Health and Human Services, CDC; 2025. https://www.cdc.gov/legionella/causes/index.html

[2] CDC. Legionnaires’ disease surveillance summary report, United States, 2020–2021. Atlanta, GA: US Department of Health and Human Services, CDC; 2025. https://www.cdc.gov/legionella/health-depts/surv-reporting/2020-21-report-tables/2020-21-surv-report-508.pdf

[3] CDC. Drinking water: about choosing home water filters. Atlanta, GA: US Department of Health and Human Services, CDC; 2024. https://www.cdc.gov/drinking-water/prevention/about-choosing-home-water-filters.html

[4] CDC. Infant and toddler nutrition: infant formula preparation and storage. Atlanta, GA: US Department of Health and Human Services, CDC; 2026. https://www.cdc.gov/infant-toddler-nutrition/formula-feeding/preparation-and-storage.html

